# Correlation between the thyroid computed tomography value and thyroid function in hyperthyroidism: a retrospective study

**DOI:** 10.1007/s12149-024-01938-0

**Published:** 2024-05-24

**Authors:** Haruna Iwanaga, Naotoshi Fujita, Shinji Abe, Shinji Naganawa, Katsuhiko Kato

**Affiliations:** 1 Department of Radiological Technology, Nagoya University Hospital, Nagoya, Japan; 2 Department of Radiological and Medical Laboratory Sciences, Department of Integrated Health Science, Nagoya University Graduate School of Medicine, Nagoya, Japan; 3 Functional Medical Imaging, Biomedical Imaging Sciences, Division of Advanced Information Health Sciences, Department of Integrated Health Sciences, Nagoya University Graduate School of Medicine, Nagoya, Japan; 4 1-20, Daikominami 1‑Chome, Higashi-Ku, Nagoya, 461-8673 Japan

**Keywords:** Graves, Disease, Plummer disease, CT value, Radioiodine therapy, Effective half-life, Thyroid

## Abstract

**Objective:**

Radioiodine (I-131) therapy for hyperthyroidism is a well-established and safe treatment option. This study aimed to investigate the relationship between the computed tomography (CT) value and the function and volume of the thyroid gland by identifying the factors that induce changes in the CT value of patients with hyperthyroidism.

**Methods:**

This retrospective study evaluated 38 patients with Graves’ disease and 10 patients with Plummer disease. To obtain the mean CT value and volume of the thyroid gland, the entire thyroid gland was set as the region of interest. A test dose of 3.7 MBq I-131 was administered before initiating I-131 therapy, and the radioiodine uptake (RIU) rate was assessed after 3, 24, 96, and 168 h. An approximate curve was plotted based on the RIU values obtained, and the effective half-life (EHL) was calculated. The correlation between the mean CT value and the volume of the thyroid gland, 24-h RIU, EHL, and the free triiodothyronine (FT3), free thyroxine (FT4), thyroid-stimulating hormone (TSH), and TSH receptor antibody (TRAb) levels was evaluated.

**Results:**

The CT value exhibited a significant positive correlation with EHL in patients with Graves’ disease (r = 0.62, *p* < 0.0001) as well as patients with Plummer disease (r = 0.74, *p* < 0.05). However, it did not display any correlation with the remaining parameters.

**Conclusion:**

The CT value is significantly correlated with EHL, suggesting that it reflects thyroid function and is mainly related to the factors associated with iodine discharge.

## Introduction

Iodine is stored in the thyroid gland for the synthesis of thyroid hormones. Therefore, thyroid glands, which contain a large amount of iodine with a high atomic number, tend to exhibit a relatively high computed tomography (CT) value. The normal thyroid CT value is approximately 110 Hounsfield units (HU) [1, 2]. In contrast, the CT value of patients with Graves' disease, a form of hyperthyroidism, is lower than the normal CT value [2]. Although the CT value may reflect thyroid function [1, 3–6]the relationship between the CT value and thyroid function has not yet been fully investigated, partly due to concerns regarding radiation exposure to patients.

The main treatment options for hyperthyroidism include medication (such as beta-blockers or antithyroid drugs), radioiodine therapy (iodine-131), and thyroidectomy [7]. Among these, I-131 therapy stands as a well-established treatment with recognised effectiveness [8, 9]. In I-131 therapy, factors such as the radioiodine uptake rate (RIU), the effective half-life (EHL), and the thyroid volume significantly influence the effectiveness of the treatment [10–12]. Therefore, it is important to evaluate thyroid function and volume beforehand and administer an appropriate amount of I-131. However, measuring the iodine uptake rate and the effective half-life is a cumbersome process, requiring the intake of I-131 for testing purposes and multiple measurements [12]. If the thyroid CT value is determined to be related to thyroid function and morphology, it could potentially serve as an alternative to the complex methods required for thyroid function evaluation.

At our hospital, plain CT imaging is conducted to measure thyroid volume, allowing us to retrospectively examine the thyroid CT value using these images. In this study, we investigated the relationship between thyroid function and volume and the thyroid CT value in patients with Graves’ disease and Plummer disease to determine whether the thyroid CT value reflects thyroid function and volume.

## Patients and methods

### Patients

Thirty-eight patients with Graves’ disease (male, n = 6; female, n = 32; age, 42.1 ± 13.7 years) and 10 patients with Plummer disease (male, n = 3; female, n = 7; age, 59.1 ± 14.5 years) who received their first I-131 therapy between March 2018 and December 2019 were included in this retrospective analysis.

### Treatment schedule

**Fig. 1:**
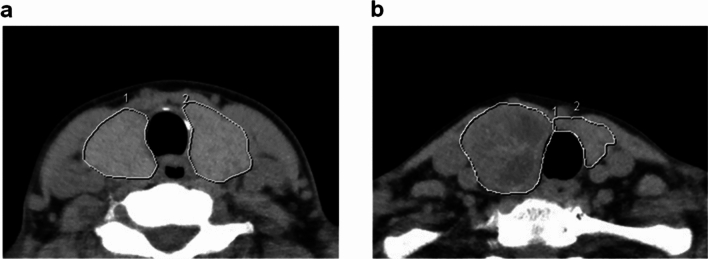
Regions of interest superimposed on the thyroid gland on the computed tomography images.The sum of the thyroid volume obtained from each slice was denoted as the total thyroid volume **a** Graves’ disease, **b** Plummer disease

The administration of antithyroid medication and potassium iodide was discontinued 2 weeks before I-131 therapy. The free triiodothyronine (FT3), free thyroxine (FT4), thyroid-stimulating hormone (TSH), and TSH receptor antibody (TRAb) levels were measured once before treatment. FT3, FT4, and TSH levels were determined using a CLEA kit (Abbott Diagnostic Medical, Chicago, IL, USA; normal range: FT3, 1.68–3.67 pg/mL; FT4, 0.70–1.48 ng/dL; TSH, 0.35–4.94 μIU/mL). TRAb levels were determined using an ECLES Kit (Roche Diagnostic Medical, Basel, Switzerland; (normal range: < 2.0 IU/L). All patients with Graves’ and Plummer disease who received I-131 therapy at our hospital underwent non-contrast CT scanning of the neck (Aquilion PRIME SP; Canon Medical Systems, Tokyo, Japan) for thyroid volumetry. The CT imaging conditions were as follows: tube voltage, 120 kVp; tube current, determined via an automatic exposure mechanism. CT images were reconstructed with a slice thickness of 5 mm, and a nuclear medicine specialist manually placed the region of interest (ROI) over the entire thyroid gland on the CT images (Fig. 1). The thyroid volume was determined using the area of the thyroid gland and the slice thickness in each slice, and the sum of the thyroid volumes obtained from all slices was used as the thyroid volume. The average CT value within the ROI placed over the entire thyroid gland was calculated (Fig. 1). The average CT value for the entire thyroid gland was calculated, excluding slices showing calcification in the thyroid gland or obvious changes in the CT value due to artefacts. RIU measurements were performed as follows. A test dose of 3.7 MBq I-131 (RADIOCAP; FUJIFILM Toyama Chemical Co., Ltd., Tokyo, Japan) was orally administered before initiation of I-131 therapy, and the RIUs of the thyroid gland were measured after 3, 24, 96, and 168 h. A thyroid uptake system (AZ-800; Anzai Medical, Tokyo, Japan) equipped with NaI crystals was used to acquire the measurements. Before measurements in patients, a neck phantom (ORINS Standard thyroid uptake phantom; Abbott) was evaluated. The I-131 capsule (activity = 3.7 MBq) was placed in the phantom and counts were taken (S). Background measurements were performed with the neck phantom shielded by a lead plate (B_1). Next, the thyroid gland in patients was measured twice, once without shielding (T) and once with shielding by a lead plate (B_2). From each measurement, RIU was calculated using Eq. 1.

1 $$\begin{array}{c}RIU=\frac{\left(T-{B}_{2}\right)}{\left(S-{B}_{1}\right)}\times 100.\end{array}$$

**Fig. 2:**
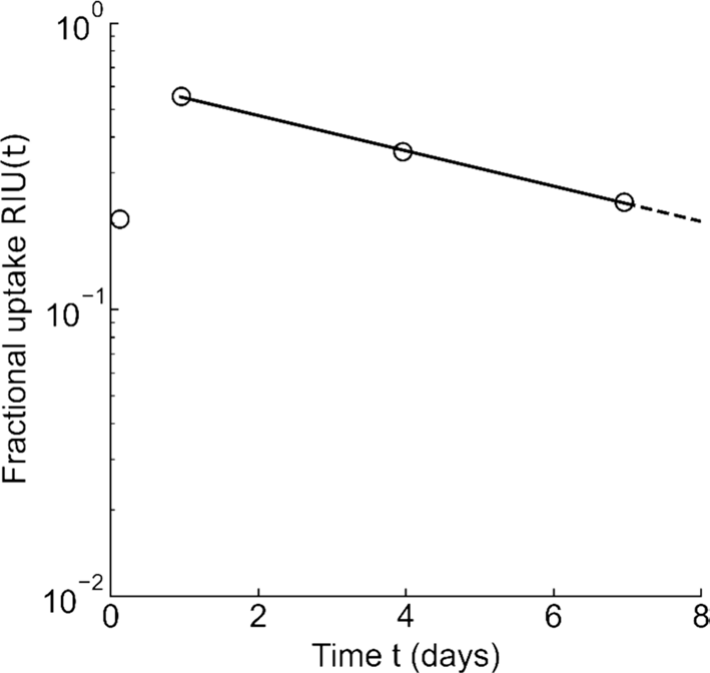
EHL calculated by approximating the RIU (circles) after 24 h as a mono-exponential function (solid line). *EHL* effective half-life, *RIU* radioiodine uptake

The 24-h RIU was calculated by correcting for attenuation so that the total count in the thyroid region was equal to the count at the time of administration. EHL was calculated by exponential approximation of the ratio of counts at each time point, obtained by Eq. 1 (Fig. 2). Because thyroid RIU generally reaches its maximum at around 24 h, EHL was calculated using RIU after 24, 96, and 168 h, when the thyroid gland was in the excretory phase. The CT imaging and RIU and EHL measurements were performed from 1 month to 1 week before the I-131 therapy. Blood tests were performed on the day of I-131 therapy before I-131 administration.

### Statistical analysis

Data were analysed using the statistical software EZR [13]. Pearson’s correlation and linear regression analyses were conducted using the mean CT value of the respective tracers. Regression analysis was conducted using the parameters with significant correlations, and regression equations were calculated. Statistical significance was set at *p* < 0.05.

## Results

Table 1 summarises the details of patients with Graves’ disease, including the CT value, thyroid volume, RIU, EHL, and the FT3, FT4, TSH, and TRAb levels.

**Table 1:** Baseline characteristics of patients with Graves’ disease and Plummer disease

	Graves’ disease (n = 38)	Plummer disease (n = 10)
Age (years)	42.1 ± 13.67	59.1 ± 14,5
Thyroid volume (mL)	85.3 ± 55.1	78.7 ± 54.4
24-h RIU (%)	73.7 ± 18.8	50.0 ± 17.7
EHL (days)	6.4 ± 1.2	6.7 ± 1.1
TSH (µIU/mL)	0.4 ± 0.7	0.01 ± 0.03
FT3 (pg/mL)	10.3 ± 6.8	4.9 ± 1.5
FT4 (ng/dL)	2.3 ± 1.1	0.4 ± 1.7
TRAb (IU/L)	14.2 ± 17.5	-
Thyroid CT value (HU)	53.3 ± 10.4	50.9 ± 11.7

Data are expressed as mean ± standard deviation. *RIU* radioiodine uptake, *EHL* effective half-life, *TSH* thyroid-stimulating hormone, *FT3* free triiodothyronine, *FT4* free thyroxine, *TRAb* TSH receptor antibody

**Fig. 3:**
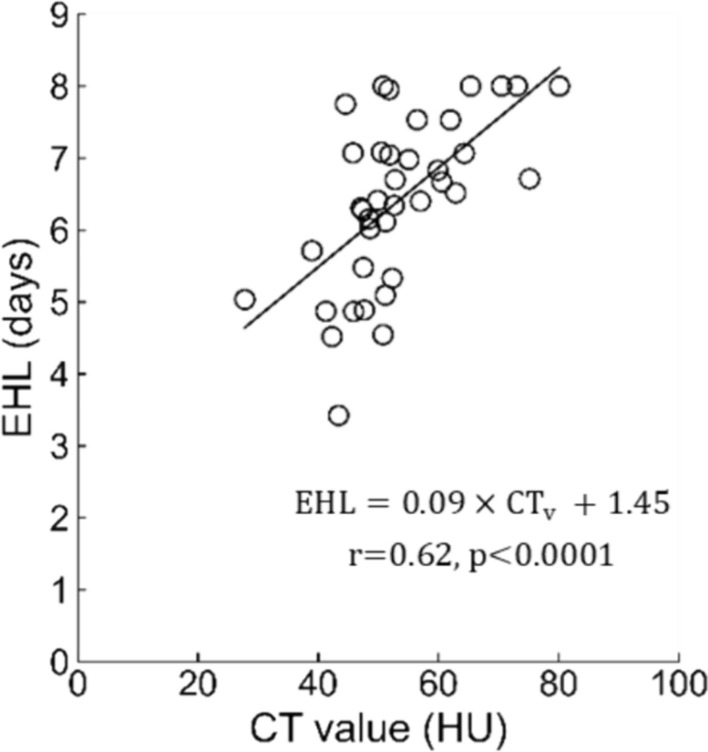
Relationship between the thyroid CT value and EHL in patients with Graves’ disease. *CT value* computed tomography value, *EHL* effective half-life

The CT value exhibited a significant positive correlation with EHL in patients with Graves’ disease (r = 0.62, *p* < 0.0001) (Fig. 3). The regression equation was as follows: EHL = 0 0.09 × CT value + 1.45.

**Fig. 4:**
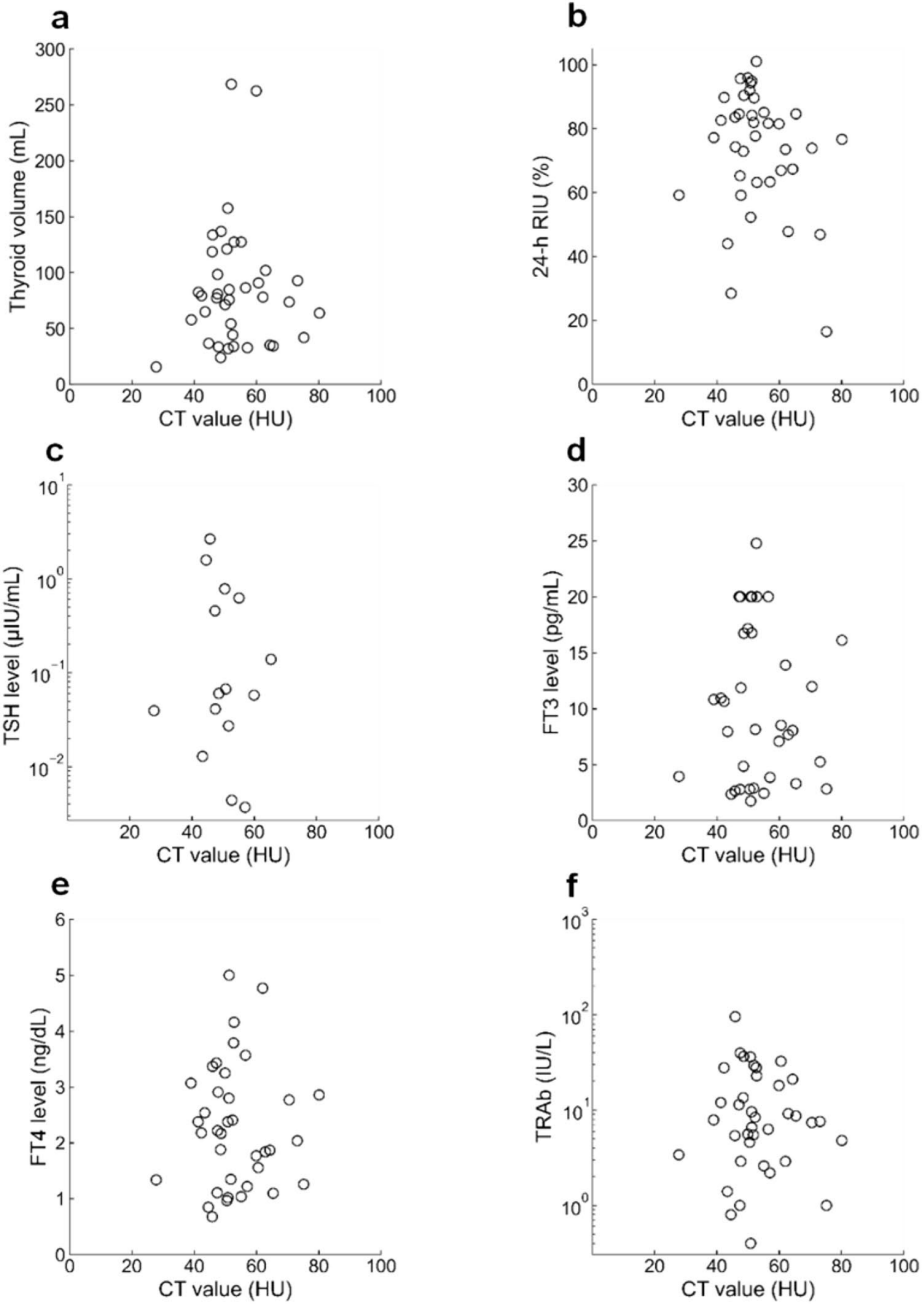
Relationship between thyroid CT value and five factors in patients with Graves’ disease. **a** Thyroid volume, **b** 24-h RIU, **c** TSH, **d** FT3, **e** FT4, **f** TRAb levels. *CT value* computed tomography value, *RIU* radioiodine uptake, *TSH* thyroid-stimulating hormone, *FT3* free triiodothyronine, *FT4* free thyroxine, *TRAb* TSH receptor antibody

The CT value exhibited no correlation with the thyroid volume (r = 0.04, *p* = 0.82), 24-h RIU (r =  − 0.23, *p* = 0.19), and FT3 (r =  − 0.02, *p* = 0.90), FT4 (r = 0.01, *p* = 0.93), TSH (r =  − 0.17, *p* = 0.553) levels, and TRAb levels (r =  − 0.14, p = 0.41) (Fig. 4). As the FT3 and FT4 levels exceeded the upper limit of measurement (FT3, 30 pg/mL; FT4, 6 ng/dL) in two and one patients, respectively, the correlation coefficients were calculated after excluding each case. Similarly, the correlation coefficients were calculated after excluding 23 patients with TSH levels below the lower limit of measurement (0.003 µIU/mL).

**Fig. 5:**
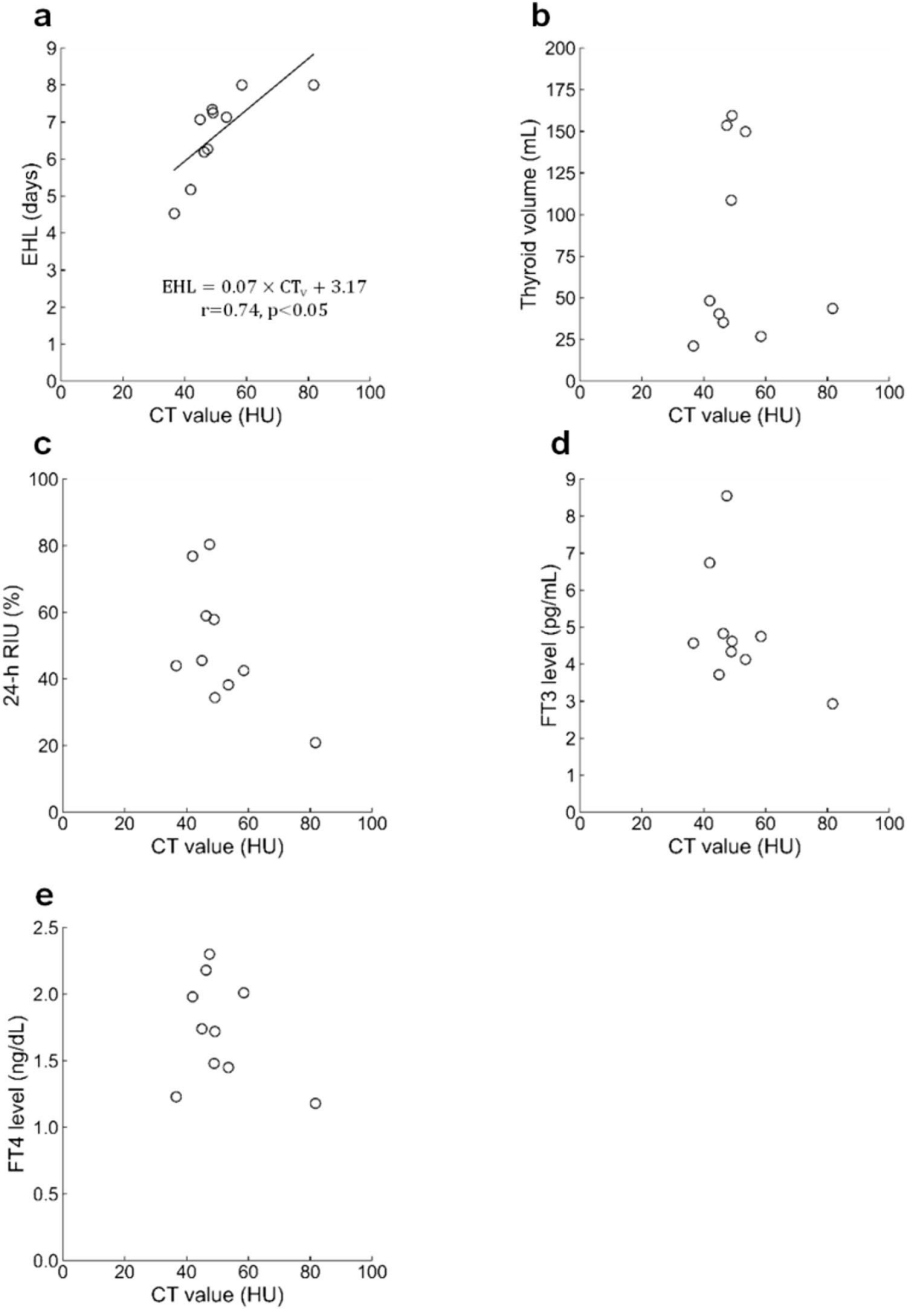
Relationship between thyroid CT value and five factors in patients with Plummer disease. **a** EHL, **b** Thyroid volume, **c** 24-h RIU, **d** FT3, **e** FT4. *CT value* computed tomography value, *EHL* effective half-life, *RIU* radioiodine uptake, *FT3* free triiodothyronine, *FT4* free thyroxine

In addition, the CT value displayed a significant positive correlation (r = 0.74, *p* < 0.05) with EHL in patients with Plummer disease (Fig. 5). The TSH levels were below the detection limit in nine of the 10 patients. All patients tested negative for TRAb. The regression equation was EHL = 0.07 × CT value + 3.17. Moreover, the CT value exhibited no correlation with the thyroid volume (r =  − 0.04, p = 0.92), 24-h RIU (r =  − 0.60, p = 0.07), and FT3 (r =  − 0.45, p = 0.20) and FT4 (r =  − 0.33, p = 0.35) levels.

## Discussion

The thyroid gland comprises numerous follicles, each containing thyroid follicular cells arranged in a circular pattern surrounding a colloid containing thyroid hormones produced from thyroglobulin and iodide. The secretion of the thyroid hormone is regulated by TSH [7]. On CT images, the CT value of the thyroid gland is approximately 100–110 HU, higher than that of soft tissues [1, 2]. This elevation in the CT value may be attributed to the presence of several colloids containing iodine in the thyroid gland. Notably, a strong correlation has been reported between the iodine concentration in the thyroid tissue and the CT value [1]. In the present study, the CT value exhibited a significant positive correlation with EHL, which is the rate of iodine accumulation or elimination from the thyroid gland. If the EHL is short and the duration from RIU to elimination is rapid, iodine is not stored in the thyroid tissue for a long duration. Therefore, patients with low CT values are likely to have a higher RIU rate for elimination in the thyroid gland. The iodine concentration in the thyroid gland is strongly correlated with the CT value; however, factors other than iodine concentration can also affect the CT value. Imanishi et al. reported that a decrease in the CT value indicates a decrease in the colloidal area associated with a decrease in iodine concentration within the colloid and an increase in the ratio of follicular cells and interstitial structures to the total thyroid gland [5]. Han et al. reported that the CT value is correlated with the standardised uptake value on positron emission tomography images, and that the CT value is correlated with inflammation in the thyroid tissue [4]. The CT values of follicular cells and interstitial tissue are comparable with those of soft tissue, resulting in lower CT values than those for normal thyroid tissue. A lower CT value may also reflect an increase in the ratio of follicular cells and interstitial tissue to total thyroid tissue.

In addition, the CT value is correlated with EHL and reflects the iodine metabolic capacity of the thyroid gland. EHL is an index required for determining the administered radioactivity of I-131 therapy. EHL should be measured at least twice within 8 days of administration of the I-131 test dose and repeated three or more times for accurate measurement. A fixed value is sometimes used to estimate the absorbed dose for I-131 therapy, as the measurement of the EHL is complicated. However, because calculating the absorbed dose with EHL as a fixed value can lead to errors, the EANM guidelines do not recommend determining radioactivity without considering thyroid volume and iodine kinetics [12]. Moreover, the CT value could be used to estimate EHL and optimise the thyroid absorbed dose if EHL is not measured, as the CT value is correlated with EHL. However, the extent to which the use of the CT value improves the cure rate remains to be determined in future studies.

TRAb was not correlated with the CT value in this study. TRAb is a TSH receptor antibody that not only inhibits the TSH receptor but also continuously stimulates it to overproduce thyroid hormones. Therefore, it has been reported that TRAb is related to the rate of iodine metabolism, and that high TRAb levels tend to have shorter EHL [14]. Because the relationship between the thyroid CT value and EHL was demonstrated in this study, a relationship with TRAb is possible. However, the efficacy of TRAb has also been reported to change with age [15], and its relationship with thyroid-stimulating antibody (TSAb), which is reportedly a more specific parameter for Graves’ disease than is TRAb [16, 17], should also be investigated.

The present study has some limitations. First, the CT images of the patients were obtained before the initiation of treatment; however, the timing differed from that of the blood tests by approximately 1 week to 1 month. Changes, such as iodine restriction or the withdrawal of antithyroid medication, occurred in some patients during this period. However, no significant relationship with thyroid hormones was observed in the present study, as the thyroid hormones are sensitive to medication. Previous studies have reported that the CT value is weakly correlated with TSH and FT4 [4, 6]. Therefore, the timing of CT imaging and blood sampling should be aligned to accurately determine the correlation between the thyroid hormone levels and the CT value. Second, the CT value in patients with Plummer disease was calculated by including the normal areas of the thyroid gland. The CT value calculated using this measurement method was correlated with EHL. However, as the number and size of nodules may affect the CT value in different patients, it is necessary to separate nodule areas from normal thyroid areas and calculate the CT value to confirm their relationship with thyroid function. Finally, although the ROIs were set for the entire thyroid gland in the present study, some patients had low CT values, and some CT values were almost equivalent to those of the surrounding soft tissues. Determining the thyroid contour in such patients was difficult, which may have contributed to errors in the calculation of the CT value. As the ROIs were set by a single nuclear medicine specialist in the present study, inter-examiner errors were not examined. In addition, artefacts may have appeared owing to the influence of the clavicle and jaw, which may have changed the CT value. Such effects are considered to be one of the factors causing errors in the calculation of the CT value.

## Conclusions

This study investigated the relationship between the CT value and the function and volume of the thyroid gland by exploring the factors related to the changes in the CT value in patients with hyperthyroidism. The CT value exhibited a significant correlation with EHL, suggesting that the CT value reflects thyroid function and is mainly related to the factors associated with iodine discharge.

## Data Availability

The datasets used and/or analysed during the current study are available from the corresponding author upon reasonable request.

## List of abbreviations

CT: Computed tomography
EANM: European association of nuclear medicine
EHL: Effective half-life
FT3: Free triiodothyronine
FT4: Free thyroxine
HU: Hounsfield units
I-131: Radioiodine
RIU: Radioiodine uptake
TRAb: TSH receptor antibody
TSH: Thyroid-stimulating hormone
